# Genetic impact of methylenetetrahydrofolate reductase (*MTHFR*) polymorphism on the susceptibility to colorectal polyps: a meta-analysis

**DOI:** 10.1186/s12881-019-0822-y

**Published:** 2019-05-30

**Authors:** Manyi Sun, Jin Zhong, Li Zhang, Songli Shi

**Affiliations:** 1 Department of Gastroenterology, Tianjin Union Medical Center, No.190, Jieyuan Road, Hongqiao District, Tianjin, 300121 People’s Republic of China; 2 Department of Radiology, Tianjin Union Medical Center, Tianjin, 300121 People’s Republic of China; 3 Department of Pathology, Tianjin Union Medical Center, Tianjin, 300121 People’s Republic of China

**Keywords:** *MTHFR*, Polymorphism, Colorectal polyps, Susceptibility

## Abstract

**Background:**

There are several studies with inconsistent conclusions regarding the association between the rs1801133 and rs1801131 polymorphisms within the *MTHFR* (methylenetetrahydrofolate reductase) gene and colorectal polyp risk. This discrepancy led us to assess the genetic impact of the two polymorphisms on the susceptibility to colorectal polyps.

**Methods:**

A meta-analysis was carried out for quantitative synthesis. According to the inclusion/exclusion criteria, we retrieved, screened and selected all published articles related to colorectal polyps and the *MTHFR* rs1801133 and rs1801131 polymorphisms. The *P* value of association test, RRs (risk ratios) and 95% CIs (confidence intervals) were mainly produced.

**Results:**

A total of twenty-three case-control studies were included from twenty-two eligible articles. Pooling the results of both rs1801133 and rs1801131 polymorphisms in the overall population suggested a nonsignificant association between colorectal polyp cases and controls, in that all *P* values in the test of association were larger than 0.05. Nevertheless, pooling results in the “UK” subgroup of rs1801131, comprising five studies (1257 cases/1407 controls), indicated an elevated risk in colorectal polyp cases in comparison with controls, under the genetic models of CC vs. AA (*P* = 0.032, RR = 1.27, 95% CIs = 1.02, 1.57) and CC vs. AA+AC (*P* = 0.036, RR = 1.27, 95% CIs = 1.02, 1.60).

**Conclusion:**

The C/C genotype of *MTHFR* rs1801131 is more likely to be a genetic risk factor for colorectal polyps in the UK region, although this finding should be verified with a larger sample size.

**Electronic supplementary material:**

The online version of this article (10.1186/s12881-019-0822-y) contains supplementary material, which is available to authorized users.

## Background

Colorectal polyps exhibit different morphologic features with flat, depressed, serrated, sessile or pedunculated shapes and are often regarded as benign protrusions of the colon and rectum mucosa [1, 2]. There are many types of colorectal polyps, such as hyperplastic polyps and adenomatous polyps [2, 3]. Despite the low malignant potential, the possible malignant change in colorectal polyps is related to the presence of colorectal cancer (CRC). For instance, some colonic polyps exist in patients with familial adenomatous polyposis (FAP) who are prone to cancer [4].

The 5,10-methylenetertahydrofolate reductase (*MTHFR*) gene is essential for the folate cycle and homocysteine metabolism [5]. rs1801133 (C677T) and rs1801131 (A1298C) are two common functional polymorphisms within the *MTHFR* gene [6, 7]. *MTHFR* rs1801133 and rs1801131 polymorphisms were reportedly associated with an enhanced risk of colorectal adenomatous polyp patients in the Korean population [8]. However, no association between the *MTHFR* rs1801133 polymorphism and colorectal adenomatous polyp susceptibility was reported in the Dutch [9] or Japanese population [10]. These findings merit a comprehensive evaluation.

To the best of our knowledge, only one reported meta-analysis [6] of the association between *MTHFR* rs1801131 and colorectal adenoma and three meta-analyses [6, 11, 12] of *MTHFR* rs1801133 and colorectal adenoma were found during the database searching. However, the conclusion remains inconsistent. Additionally, we failed to retrieve a meta-analysis specific for the association between *MTHFR* polymorphisms and the susceptibility to both hyperplastic/adenomatous polyps. Herein, we have made an attempt to better investigate the potential genetic role of *MTHFR* rs1801133 and rs1801131 polymorphisms in the risk of colorectal polyps through an updated meta-analysis.

## Methods

### Database searching and screening process

Two authors (MS and JZ) gathered the relative records through searching the databases, namely, PubMed, WOS (Web of Science), and EMBASE (Excerpta Medica Database), prior to March 2018. The PRISMA (Preferred Reporting Items for Systematic Reviews and Meta-Analyses) guidelines were followed [13]. The search terms used with the databases are shown in Additional file 1: Table S1. We independently excluded duplicate and ineligible records based on the following criteria: reviews, mouse data, case reports or trials, meta-analyses, meeting or conference abstracts, other genes, non-SNP or nonpolyp data, or missing genotype data for rs1801133 or rs1801131. Then, the remaining studies were included as eligible case-control studies.

### Data extraction and quality assessment

We carefully extracted the data from the above selected studies. The chi-squared test was applied for the calculation of the *P* value of HWE (Hardy-Weinberg Equilibrium). The included studies should provide the genotype frequency data of the control group, which also must be in line with the requirement of HWE. We summarized the main features of the included studies, such as first author name, publication year, polymorphism genotype frequency, country, ethnicity, genotyping assay, and *P* value of HWE. We also utilized quality assessment (Newcastle-Ottawa Scale, NOS) to determine the quality score of the enrolled studies. Studies with poor quality (NOS score less than five) were excluded.

### Association test

We obtained the *P*_association_, risk ratios (RRs) and 95% confidence intervals (CIs) through the association test. The *P*_heterogeneity_ value of Cochran’s Q statistic > 0.1 or I^2^ value < 50% led us to use a fixed-effects model. Six genetic models were used: allele T vs. allele C for rs1801133, allele C vs. allele A for rs1801131 (allele); TT vs. CC, CC vs. AA (homozygote); CT vs. CC, AC vs. AA (heterozygote); CT + TT vs. CC, AC + CC vs. AA (dominant); TT vs. CC + CT, CC vs. AA+AC (recessive); carrier T vs. carrier C, carrier C vs. carrier A (carrier).

### Heterogeneity source analysis

We also carried out a sensitivity analysis and subgroup analyses for all genetic models to evaluate the data stability and source of heterogeneity. Briefly, we omitted each included study in turn to acquire a group of meta-analysis estimations. The omitted study was regarded as the probable heterogeneity source if we detected an obvious alteration of RR and 95% CI value. Subgroup analyses were also carried out, taking the factors of country, ethnicity (Caucasian/Asian) and disease type (hyperplastic polyps/ adenomatous polyps) into consideration.

### Publication bias analysis

We conducted both Begg’s test (Begg’s funnel plot) and Egger’s test (Egger’s publication bias plot) to evaluate possible publication bias. The absence of a large publication bias was considered when the *P* values of Begg’s test and Egger’s test were > 0.05. STATA/SE software (StataCorp, USA) was utilized for all the above tests.

## Results

### Identification of eligible studies

**FIG. 1:**
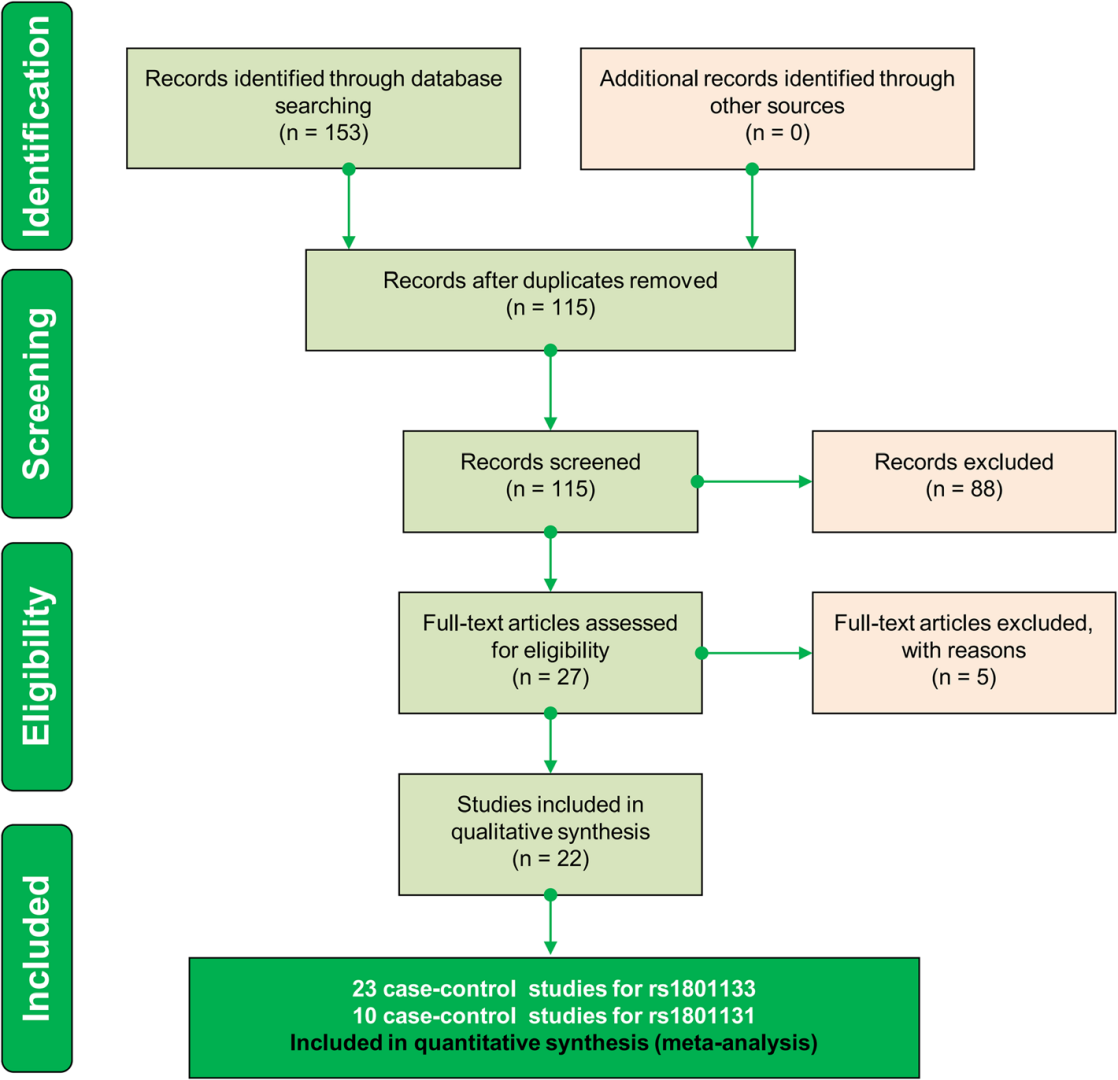
Flowchart of database searching and record screening process

We initially identified a total of 153 records by searching three databases, namely, PubMed (*n* = 22), WOS (*n* = 83), and EMBASE (*n* = 48). After excluding duplicate records, a total of 115 records were filtered by our criteria. The following 88 records were excluded: reviews (*n* = 31), mouse data (n = 4), case reports or trials (*n* = 7), meta-analyses (*n* = 6), meeting or conference abstracts (n = 8); other genes (*n* = 9), non-SNP or nonpolyp data (*n* = 23). Subsequently, twenty-seven full-text articles were evaluated for eligibility. Five articles lacked control or T/T genotype data. Finally, a total of twenty-two articles [8–10, 14–32] were selected. We listed the characteristics of eligible studies in the meta-analysis (Table 1). The genotype contributions of all controls in the studies fulfilled the principle of HWE. We found that one article contained two case-control studies, namely, the genotype distribution data in both adenomatous and hyperplastic polyps. In total, twenty-three case-control studies were ultimately included for the overall meta-analysis of *MTHFR* rs1801133, and ten case-control studies were included for that of *MTHFR* rs1801131. In addition, one study in which the TT genotype frequency of case and control groups for rs1801133 equaled zero was not included in the meta-analysis under the TT vs. CC (homozygote) and TT vs. CC + CT (recessive) models. The PRISMA-based analysis flowchart is shown in Fig. 1. None of the included studies exhibited poor quality (all NOS scores were larger than five).

**Table 1:** Main features of eligible studies for pooled analysis

First author	Year	NOS	Polymorphism	Case			Disease type	Control			Country	Ethnicity	Genotyping assay	*P*_*HWE*_
				A/A	A/B	B/B		A/A	A/B	B/B				
Al-Ghnaniem [14]	2007	7	rs1801133	22	12	1	adenomatous polyps	41	29	6	UK	Caucasian	PCR-RFLP	0.784
			rs1801133	11	3	3	hyperplastic polyps	41	29	6	UK	Caucasian	PCR-RFLP	0.784
			rs1801131	18	12	5	adenomatous polyps	47	26	3	UK	Caucasian	PCR-RFLP	0.799
			rs1801131	8	7	2	hyperplastic polyps	47	26	3	UK	Caucasian	PCR-RFLP	0.799
Ashktorab [15]	2007	6	rs1801133	18	4	0	colorectal polyps	30	5	0	USA	Caucasian	PCR-RFLP	0.649
Beckett [16]	2015	5	rs1801133	29	20	7	adenomatous polyps	88	91	18	Australia	Caucasian	PCR-RFLP	0.421
			rs1801131	28	22	6	adenomatous polyps	101	83	13	Australia	Caucasian	PCR-RFLP	0.460
Chen [17]	1998	8	rs1801133	102	126	30	adenomatous polyps	323	324	66	USA	Caucasian	PCR-RFLP	0.234
Chiang [18]	2015	7	rs1801133	44	26	0	adenomatous polyps	91	73	18	China	Asian	PCR-RFLP	0.553
de Vogel [19]	2011	6	rs1801133	947	714	135	adenomatous polyps	4463	3563	708	Norway	Caucasian	Real-time PCR	0.933
Delgado [20]	2001	8	rs1801133	6	19	7	adenomatous polyps	34	52	24	Mexico	Caucasian	PCR-RFLP	0.625
Giovannucci [21]	2003	6	rs1801133	157	168	49	adenomatous polyps	299	325	101	USA	Caucasian	PCR-RFLP	0.401
			rs1801131	186	165	24	adenomatous polyps	369	299	57	USA	Caucasian	PCR-RFLP	0.740
Goode [22]	2004	7	rs1801133	236	196	58	adenomatous polyps	259	238	67	USA	Caucasian	PCR-RFLP	0.281
Hazra [23]	2007	7	rs1801133	217	245	63	adenomatous polyps	229	232	64	USA	Caucasian	NA	0.658
			rs1801131	278	211	48	adenomatous polyps	264	219	46	USA	Caucasian	NA	0.951
Hirose [24]	2005	8	rs1801133	182	203	67	adenomatous polyps	399	496	155	Japan	Asian	PCR-RFLP	0.966
Yi [8]	2006	6	rs1801133	5	5	4	adenomatous polyps	2	4	0	Korea	Asian	PCR-RFLP	0.221
			rs1801131	10	3	1	adenomatous polyps	3	3	0	Korea	Asian	PCR-RFLP	0.414
Levine [25]	2000	7	rs1801133	256	163	52	adenomatous polyps	263	198	49	USA	Caucasian	PCR-RFLP	0.193
Lightfoot [26]	2008	8	rs1801133	135	132	41	adenomatous polyps	130	139	27	UK	Caucasian	Taqman drug metabolizing genotyping assays	0.238
			rs1801131	155	124	29	adenomatous polyps	140	130	26	UK	Caucasian	Taqman drug metabolizing genotyping assays	0.590
Marugame [10]	2000	8	rs1801133	83	92	30	adenomatous polyps	89	105	26	Japan	Asian	PCR-RFLP	0.555
Mitrou [27]	2006	7	rs1801133	405	376	87	adenomatous polyps	402	407	89	UK	Caucasian	PCR-RFLP	0.340
			rs1801131	383	375	104	adenomatous polyps	415	380	88	UK	Caucasian	PCR-RFLP	0.941
Pufulete [28]	2003	7	rs1801133	20	13	2	adenomatous polyps	41	29	6	UK	Caucasian	PCR-RFLP	0.784
			rs1801131	18	12	5	adenomatous polyps	47	26	3	UK	Caucasian	PCR-RFLP	0.799
Ulrich [29]	1999	9	rs1801133	258	219	50	adenomatous polyps	303	269	73	USA	Caucasian	PCR-RFLP	0.260
Ulrich [30]	2000	7	rs1801133	98	72	26	hyperplastic polyps	297	258	71	USA	Caucasian	PCR-RFLP	0.192
van den [9]	2005	7	rs1801133	343	346	79	adenomatous polyps	325	305	79	Netherlands	Caucasian	PCR-RFLP	0.560
Williams [31]	2013	7	rs1801133	34	48	8	adenomatous polyps	44	42	9	UK	Caucasian	PCR-RFLP	0.822
Yamaji [32]	2009	6	rs1801133	263	325	124	adenomatous polyps	219	324	120	Japan	Asian	TaqMan PCR	0.993
			rs1801131	452	228	32	adenomatous polyps	441	197	25	Japan	Asian	TaqMan PCR	0.609

*A/A* C/C genotype of rs1801133, or A/A genotype of rs1801131, *A/B* C/T genotype of rs1801133, or A/C genotype of rs1801131, *B/B* T/T genotype of rs1801133, or C/C genotype of rs1801131, *NA* not available, *PCR-RFLP* polymerase chain reaction-restriction fragment length polymorphism, *HWE* Hardy-Weinberg Equilibrium, *NOS* Newcastle-Ottawa Scale

### Pooled analysis for *MTHFR* rs1801133

**FIG. 2:**
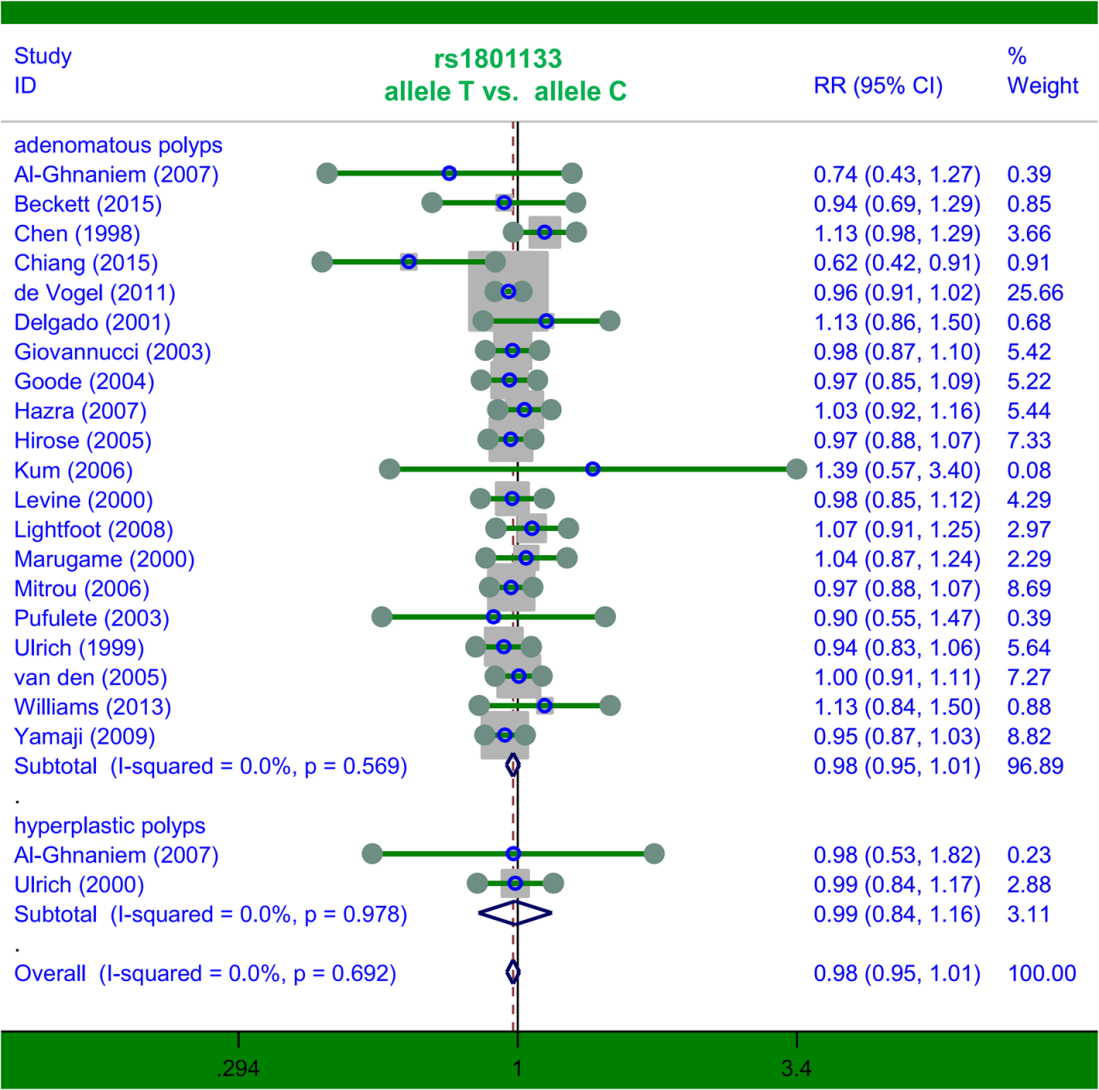
Subgroup analysis by disease type of association between *MTHFR* rs1801133 polymorphism and colorectal polyp risk under the allele T vs. allele C model

First, we carried out a meta-analysis to investigate the genetic relationship between *MTHFR* rs1801133 and colorectal polyp susceptibility. A total of twenty-three case-control studies with 8321 cases and 17,731 controls were included. As shown in Table 2, compared with the control group, no increased risk of colorectal polyps was detected in the case group under the six genetic models, namely, allele T vs. allele C (*P* value in test of association =0.156); TT vs. CC (*P* = 0.454); CT vs. CC (*P* = 0.077); CT + TT vs. CC (*P* = 0.079); TT vs. CC + CT (*P* = 0.847); carrier T vs. carrier C (*P* = 0.322). We also conducted subgroup analyses by country, ethnicity (Caucasian/Asian) and disease type (hyperplastic polyps/adenomatous polyps). A similar nonsignificant genetic relationship was observed for all the models (all *P* > 0.05, Table 2). For example, there was no significant difference between the colorectal polyp cases and negative controls in the UK subgroup under the T vs. C allele (Table 2, *P* = 0.886); TT vs. CC (*P* = 0.641); CT vs. CC (*P* = 0.351); CT + TT vs. CC (*P* = 0.511); TT vs. CC + CT (*P* = 0.436); or carrier T vs. carrier C (*P* = 0.831). In the subgroup analysis of “adenomatous polyps”, we also did not observe a statistically significant association under the allele T vs. allele C (Table 2, *P* = 0.153); TT vs. CC (*P* = 0.377); CT vs. CC (*P* = 0.113); CT + TT vs. CC (*P* = 0.098); TT vs. CC + CT (*P* = 0.696); and carrier T vs. carrier C (*P* = 0.331). We show the forest plots of the subgroup analyses based on disease type under the allele T vs. allele C model in Fig. 2. These results revealed that *MTHFR* rs1801133 does not appear to be significantly linked to susceptibility to colorectal polyps.

**Table 2:** Pooled analysis for the *MTHFR* rs1801133 polymorphism

Comparison	Subgroup	Sample size		Test of association		
		Studies	Case/control	RRs (95% CIs)	z	*P*
allele T vs. allele C	overall	23	8321/17,731	0.98 (0.95, 1.01)	1.42	0.156
	UK	6	1353/1517	0.99 (0.92, 1.07)	0.14	0.886
	USA	8	2863/4343	1.00 (0.95, 1.05)	0.14	0.890
	Japan	3	1369/1933	0.97 (0.91, 1.03)	1.03	0.301
	Caucasian	18	6868/15,610	0.99 (0.96, 1.02)	0.86	0.391
	Asian	5	1453/2121	0.95(0.90, 1.01)	1.53	0.126
	hyperplastic polyps	2	213/702	0.99 (0.84, 1.16)	0.13	0.897
	adenomatous polyps	20	8086/16,994	0.98 (0.95, 1.01)	1.43	0.153
TT vs. CC	overall	22	8317/17,696	0.97 (0.90, 1.05)	0.75	0.454
	UK	6	1353/1517	1.05 (0.85, 1.30)	0.47	0.641
	USA	7	2841/4308	1.01 (0.89, 1.14)	0.11	0.913
	Japan	3	1369/1933	0.95 (0.82, 1.11)	0.61	0.540
	Caucasian	17	6846/15,575	0.99 (0.91, 1.08)	0.31	0.760
	Asian	5	1453/2121	0.92(0.80, 1.07)	1.06	0.291
	hyperplastic polyps	2	213/702	1.13 (0.77, 1.65)	0.62	0.532
	adenomatous polyps	20	8086/16,994	0.97(0.77, 1.65)	0.88	0.377
CT vs. CC	overall	23	8321/17,731	0.97 (0.94, 1.00)	1.77	0.077
	UK	6	1353/1517	0.96 (0.89, 1.04)	0.93	0.351
	USA	8	2863/4343	0.99 (0.94, 1.04)	0.44	0.663
	Japan	3	1369/1933	0.94 (0.88, 1.01)	1.67	0.094
	Caucasian	18	6868/15,610	0.98 (0.95, 1.01)	1.11	0.269
	Asian	5	1453/2121	0.94 (0.87, 1.00)	1.92	0.055
	hyperplastic polyps	2	213/702	0.88 (0.73, 1.07)	1.27	0.205
	adenomatous polyps	20	8086/16,994	0.98 (0.95, 1.01)	1.58	0.113
CT + TT vs. CC	overall	23	8321/17,731	0.98 (0.95, 1.00)	1.76	0.079
	UK	6	1353/1517	0.98 (0.91, 1.06)	0.66	0.511
	USA	8	2863/4343	0.99 (0.95, 1.04)	0.33	0.743
	Japan	3	1369/1933	0.96 (0.91, 1.01)	1.53	0.125
	Caucasian	18	6868/15,610	0.98 (0.96, 1.01)	1.08	0.280
	Asian	5	1453/2121	0.95 (0.90, 1.00)	1.95	0.052
	hyperplastic polyps	2	213/702	0.94 (0.80, 1.09)	0.82	0.414
	adenomatous polyps	20	8086/16,994	0.98 (0.95, 1.00)	1.65	0.098
TT vs. CC + CT	overall	22	8317/17,696	0.99 (0.92, 1.07)	0.19	0.847
	UK	6	1353/1517	1.09 (0.87, 1.36)	078	0.436
	USA	7	2841/4308	1.02 (0.89, 1.16)	0.23	0.822
	Japan	3	1369/1933	1.01 (0.86, 1.18)	0.08	0.934
	Caucasian	17	6846/15,575	1.00 (0.91, 1.09)	0.07	0.944
	Asian	5	1453/2121	0.98 (0.83, 1.15)	0.28	0.780
	hyperplastic polyps	2	213/702	1.23 (0.83, 1.84)	1.04	0.299
	adenomatous polyps	20	8086/16,994	0.98(0.91, 1.84)	0.39	0.696
carrier T vs. carrier C	overall	23	8321/17,731	0.99 (0.96, 1.01)	0.99	0.322
	UK	6	1353/1517	0.99 (0.92, 1.07)	0.21	0.831
	USA	8	2863/4343	1.00 (0.95, 1.05)	0.15	0.883
	Japan	3	1369/1933	0.98 (0.91, 1.05)	0.69	0.491
	Caucasian	18	6868/15,610	0.99 (0.96, 1.02)	0.64	0.523
	Asian	5	1453/2121	0.97 (0.90, 1.03)	0.99	0.322
	hyperplastic polyps	2	213/702	0.98 (0.82, 1.16)	0.26	0.793
	adenomatous polyps	20	8086/16,994	0.99(0.96, 1.02)	0.97	0.331

*RRs* Risk ratios, *CIs* Confidence intervals

### Pooled analysis for *MTHFR* rs1801131

**FIG. 3:**
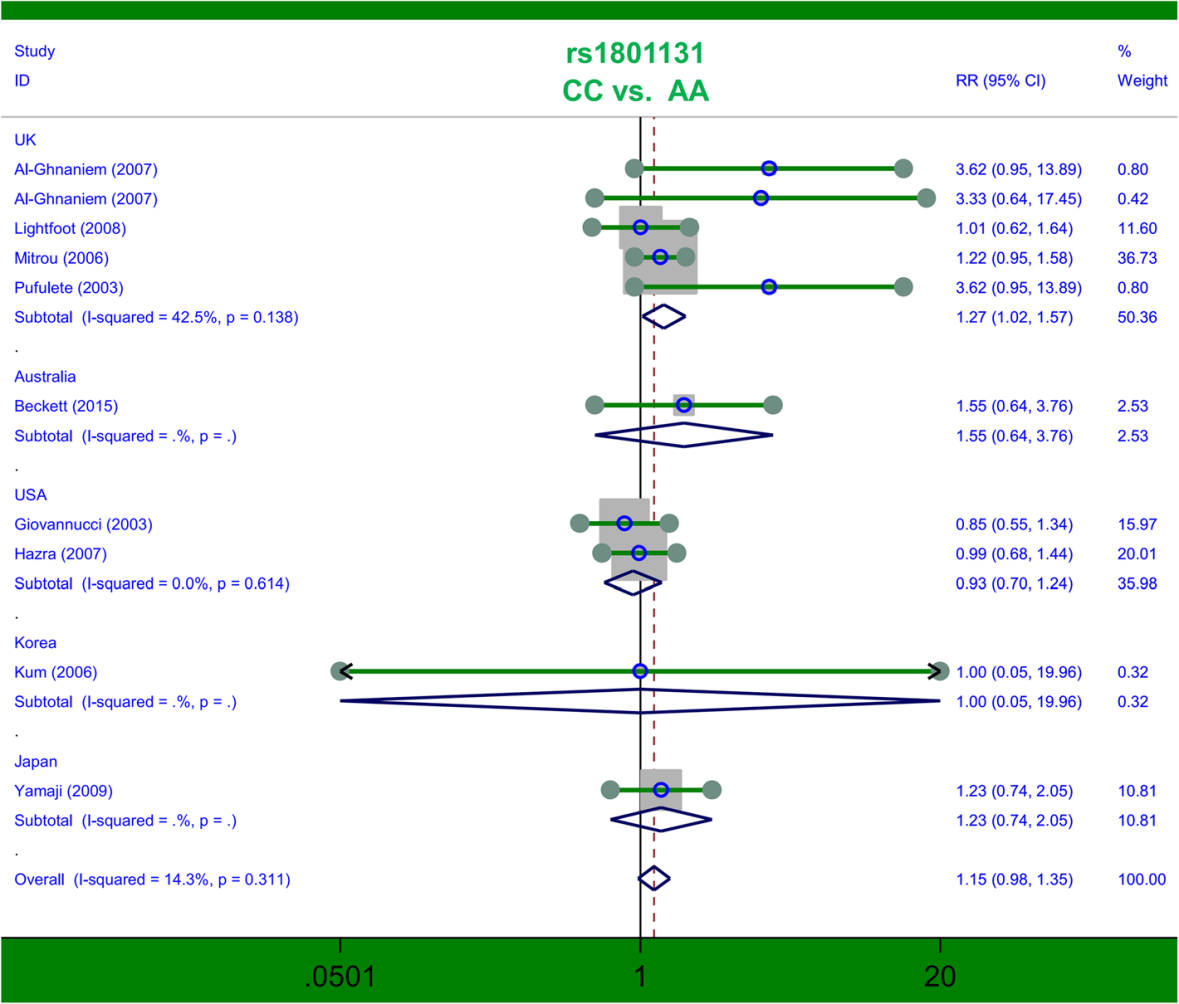
Subgroup analysis by country of association between *MTHFR* rs1801131 polymorphism and colorectal polyp risk under the CC vs. AA model

**FIG. 4:**
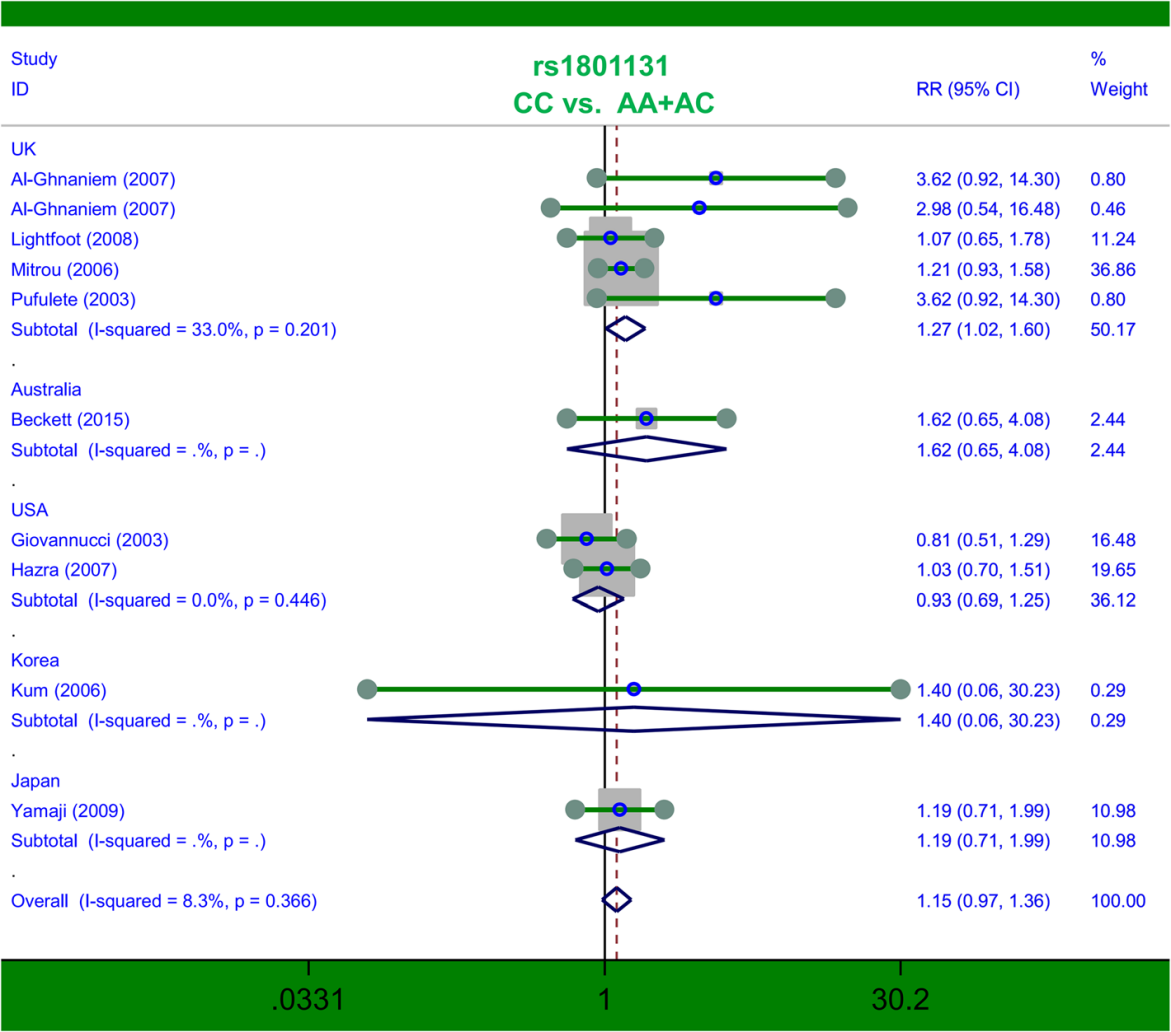
Subgroup analysis by country of association between *MTHFR* rs1801131 polymorphism and colorectal polyp risk under the CC vs. AA+AC model

Next, ten studies containing 2951 cases and 3527 controls were included in the meta-analysis of *MTHFR* rs1801131. Pooled analysis in the overall population (Table 3) indicated a null association under all genetic models (all *P* > 0.05). The results of the subgroup analysis for the UK, containing five studies of 1257 cases/1407 controls, suggested an increased risk in cases of colorectal polyps compared with controls under the genetic models of CC vs. AA (*P* = 0.032, RR = 1.27, 95% CIs = 1.02, 1.57) and CC vs. AA+AC (*P* = 0.036, RR = 1.27, 95% CIs = 1.02, 1.60). We showed the related forest plots in Figs. 3 and 4. Nevertheless, no difference between cases and controls was observed in other subgroup meta-analyses (all *P* > 0.05, Table 3). For example, no increased or decreased risk of adenomatous polyps in cases was detected, compared with controls, under the allele C vs. allele A (Table 3, *P* = 0.138); CC vs. AA (*P* = 0.114); AC vs. AA (*P* = 0.576); AC + CC vs. AA (*P* = 0.303); CC vs. AA+AC (*P* = 0.122); or carrier T vs. carrier C (*P* = 0.376). Thus, the C/C genotype of the *MTHFR* rs1801131 polymorphism may be related to an enhanced colorectal polyp risk in the UK population.

**Table 3:** Pooled analysis for the *MTHFR* rs1801131 polymorphism

Comparison	Subgroup	Sample size		Test of association		
		Studies	case/control	RRs (95% CIs)	z	*P*
allele C vs. allele A	overall	10	2951/3527	1.05 (0.99, 1.11)	1.60	0.109
	UK	5	1257/1407	1.08 (0.99, 1.17)	1.79	0.073
	Caucasian	8	2225/2858	1.04 (0.98, 1.10)	1.22	0.222
	adenomatous polyps	9	2934/3451	1.04 (0.99, 1.10)	1.48	0.138
CC vs. AA	overall	10	2951/3527	1.15 (0.98, 1.35)	1.69	0.091
	UK	5	1257/1407	1.27 (1.02, 1.57)	2.14	**0.032**
	Caucasian	8	2225/2858	1.14 (0.96, 1.35)	1.50	0.133
	adenomatous polyps	9	2934/3451	1.14 (0.97, 1.34)	1.58	0.114
AC vs. AA	overall	10	2951/3527	1.02 (0.96, 1.08)	0.63	0.528
	UK	5	1257/1407	1.02 (0.93, 1.11)	0.39	0.698
	Caucasian	8	2225/2858	1.01 (0.95, 1.07)	0.25	0.805
	adenomatous polyps	9	2934/3451	1.02 (0.96, 1.08)	0.56	0.576
AC + CC vs. AA	overall	10	2951/3527	1.03 (0.98, 1.08)	1.13	0.258
	UK	5	1257/1407	1.04 (0.97, 1.12)	1.08	0.279
	Caucasian	8	2225/2858	1.02 (0.97, 1.08)	0.72	0.471
	adenomatous polyps	9	2934/3451	1.03 (0.98, 1.08)	1.03	0.303
CC vs. AA + AC	overall	10	2951/3527	1.15 (0.97, 1.36)	1.64	0.100
	UK	5	1257/1407	1.27 (1.02, 1.60)	2.10	**0.036**
	Caucasian	8	2225/2858	1.14 (0.96, 1.36)	1.49	0.135
	adenomatous polyps	9	2934/3451	1.14 (0.97, 1.35)	1.55	0.122
carrier C vs. carrier A	overall	10	2951/3527	1.03 (0.97, 1.09)	0.96	0.336
	UK	5	1257/1407	1.04 (0.96, 1.14)	1.00	0.318
	Caucasian	8	2225/2858	1.02 (0.96, 1.09)	0.68	0.499
	adenomatous polyps	9	2934/3451	1.03 (0.97, 1.09)	0.88	0.376

*PB* Population-based control, *HB* Hospital-based control, *RRs* Risk ratios, *CIs* Confidence intervals

Bold entries are significant

### Heterogeneity, publication bias and sensitivity analysis

**FIG. 5:**
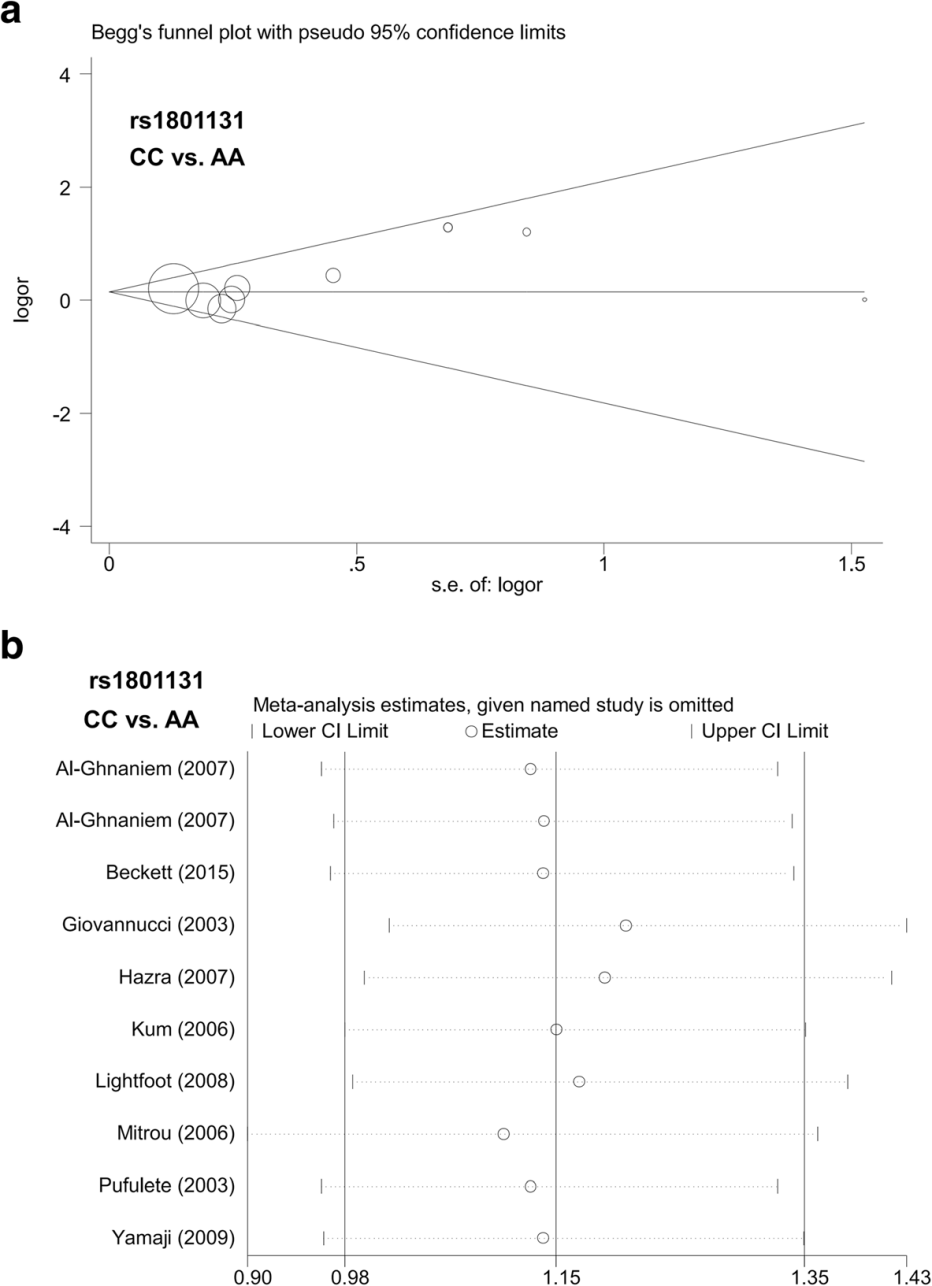
Begg’s funnel plot and sensitivity analysis for *MTHFR* rs1801131 polymorphism and colorectal polyp risk under the CC vs. AA model. **a** Begg’s funnel plot; **b** Sensitivity analysis

In addition, we evaluated the between-study heterogeneity and did not detect remarkable heterogeneity in any of the above comparisons (Table 4, all I^2^ < 50.0%, *P* value of heterogeneity > 0.1). Thus, a fixed-effects model was applied. We also conducted both Begg’s test and Egger’s test to assess the presence of publication bias. As shown in Table 4, the *P* values of Begg’s test and Egger’s test were larger than 0.05 in all genetic models, indicating the absence of large publication bias. We showed Begg’s funnel plot and the association between the *MTHFR* rs1801131 polymorphism and colorectal polyp risk under the CC vs. AA model in Fig. 5a. Additionally, similar pooled RRs were detected in our sensitivity analysis under other genetic models (Fig. 5b for CC vs. AA model of *MTHFR* rs1801131; other data not shown), suggesting the reliability of pooling outcomes.

**Table 4:** The assessment of heterogeneity and publication bias

polymorphism	Comparison	I^2^	*P* value	Model	Begg’s test		Egger’s test	
					z	*P*	t	*P*
rs1801133	allele T vs. allele C	0.0%	0.736	Fixed	0.69	0.492	0.46	0.651
	TT vs. CC	0.0%	0.799	Fixed	0.90	0.367	0.75	0.463
	CT vs. CC	0.0%	0.705	Fixed	0.79	0.428	−0.41	0.685
	CT + TT vs. CC	0.0%	0.725	Fixed	0.11	0.916	−0.02	0.984
	TT vs. CC + CT	0.0%	0.790	Fixed	0.73	0.463	0.70	0.492
	carrier T vs. carrier C	0.0%	0.999	Fixed	0.32	0.751	0.27	0.787
rs1801131	allele C vs. allele A	9.6%	0.354	Fixed	1.16	0.245	1.41	0.195
	CC vs. AA	14.3%	0.311	Fixed	1.52	0.128	1.96	0.085
	AC vs. AA	0.0%	0.800	Fixed	0.45	0.655	−0.25	0.807
	AC + CC vs. AA	0.0%	0.623	Fixed	1.34	0.180	0.64	0.541
	CC vs. AA+AC	8.3%	0.366	Fixed	1.52	0.128	2.17	0.061
	carrier C vs. carrier A	0.0%	0.918	Fixed	0.98	0.325	1.04	0.327

## Discussion

Several meta-analyses have reported the role of *MTHFR* polymorphisms in the susceptibility to colorectal cancer (CRC) and adenoma. For example, in 2005, Kono, S. and colleague included a total of 16 case-control studies for a meta-analysis on the genetic relationship between *MTHFR* rs1801133 polymorphism and the risk of colorectal cancer and reported the potential role of the TT genotype in reduced CRC susceptibility [11]. In 2007, Huang, Y. et al. performed another meta-analysis to report that *MTHFR* rs1801133 and rs1801131 polymorphisms may confer reduced susceptibility to CRC patients [6]. In 2011, Zacho, J. et al. enrolled 75,000 cases and 93,000 controls for a meta-analysis of the association between the *MTHFR* rs1801133 polymorphism and overall cancer susceptibility and found that the TT genotype of *MTHFR* rs1801133 was associated with a decreased risk in CRC patients with lifelong hyperhomocysteinemia and hence hypomethylation [33]. Recently, data from another updated meta-analysis with 37,049 cases and 52,444 controls from 91 case-control studies suggested that the *MTHFR* rs1801133 polymorphism was related to a reduced risk of CRC, particularly in the Asian population [34]. These data supported the protective effect of *MTHFR* polymorphism, especially rs1801133, on CRC risk. However, inconsistent results regarding the role of the *MTHFR* polymorphism in the risk of colorectal adenoma were observed in the quantitative synthesis.

Meta-analysis of Huang, Y. et al. revealed that *MTHFR* rs1801133 and rs1801131 polymorphisms may have no increasing or decreasing effect on the risk of colorectal adenoma patients [6]. In addition, Edwards, T. L. and colleagues included 2551 colorectal adenoma cases and 3285 controls in the Caucasian population and performed genome-wide association studies (GWASs) to identify potential susceptibility factors, but *MTHFR* polymorphisms did not reach a genome-wide significant *P* value [35]. However, Kono, S. and colleagues reported that the TT genotype of the *MTHFR* rs1801133 polymorphism may be associated with high susceptibility to colorectal adenoma in patients with poor folate status [11]. In 2016, Montazeri, Z. and colleague conducted a systematic review and meta-analyses to assess the association between 37 polymorphisms within 26 genes and colorectal adenoma risk and observed the potential genetic role of *the MTHFR* rs1801133 polymorphism, but with a relatively lower statistical power [12].

In this study, we intended to reassess the role of the *MTHFR* rs1801133 polymorphism in the susceptibility to colorectal adenomas in terms of colorectal polyps by means of a meta-analysis containing twenty-three case-control studies with 8339 cases and 17,731 controls. Our findings did not show any association between the *MTHFR* rs1801133 polymorphism and the risk of colorectal adenomatous polyps or hyperplastic polyps.

Moreover, we performed another meta-analysis of ten case-control studies with 2969 cases and 3527 controls and found that the C/C genotype of the *MTHFR* rs1801131 polymorphism has a significant influence on an increased risk of colorectal polyps in the UK population. The A to C substitution in exon seven of *MTHFR* gene-induced abnormal enzymatic activity, homocysteine or folate level and DNA methylation/synthesis may be implicated in this process. It is noteworthy that, based on the requirement of meta-analysis for the enrolled case-control number, we evaluated only the subgroup analysis data with at least three case-control studies. Therefore, the subgroup analysis data for Australia, the USA, Korea, and Japan, with one or two case-control studies, exhibits very limited statistical power. We still cannot exclude the potential effect of the *MTHFR* rs1801131 polymorphism in colorectal polyp patients of other regions.

The case-control studies in our analysis were screened by fulfilling our strict selection criteria. All the studies exhibit high quality. In addition, we observed no heterogeneity in any of the Mantel-Haenszel statistics and excluded the large publication bias. Moreover, the stability of the statistical outcomes was detected by the sensitivity analysis. Nevertheless, we are also aware of several limitations. The main problem is the small sample size in the included case-control studies. For example, only one case-control study analyzed the correlation between the *MTHFR* rs1801131 polymorphism and hyperplastic polyp risk [14]. Second, only two SNPs were measured in our study. We did not study the genetic effects of other SNPs, combination with other genes, or the levels of folate, homocysteine, vitamin B12 and colorectal polyp risk. Third, hyperplastic and adenomatous polyps have complex and different etiologies. As a genetic effect of *MTHFR* rs1801133 and rs1801131 polymorphisms has been suggested in the susceptibility to colorectal cancer [6, 11, 33, 34], additional confounding factors such as smoking, drinking, age, sex, and patient features should be adjusted for further investigation of the *MTHFR* variants in the malignant conversion from colorectal polyp.

## Conclusion

Taken together, our findings conclude that *MTHFR* rs1801131, rather than rs1801133, is more likely to be associated with an increased susceptibility to colorectal polyps in the UK population. Additionally, the C/C genotype of *MTHFR* rs1801131 may confer an increased susceptibility to patients with colorectal polyps in the UK region. However, this conclusion merits further confirmation with a larger sample size.

## Additional file

Additional file 1: **Table S1.** The search terms used with the PubMed, WOS and EMBASE databases. (DOCX 30 kb)

## Abbreviations

CI: Confidence interval
CRC: Colorectal cancer
FAP: Familial adenomatous polyposis
GWAS: Genome-wide association studies
HWE: Hardy-Weinberg Equilibrium
MTHFR: Methylenetetrahydrofolate reductase
NOS: Newcastle-Ottawa Scale
RRs: Risk ratios
