# Supplementary material for: Genetic impact of methylenetetrahydrofolate reductase (MTHFR) polymorphism on the susceptibility to colorectal polyps: a meta-analysis
Source: BMC Med Genet. 2019 May 30;20:94. doi: 10.1186/s12881-019-0822-y (PMC6543585; doi:10.1186/s12881-019-0822-y)
Supplement: Supplementary file 1 — Table S1. The search terms used with the PubMed, WOS and EMBASE databases. (DOCX 30 kb) [file 12881_2019_822_MOESM1_ESM.docx]

Table S1 The search terms of Pubmed, WOS and EMBASE databases

**Pubmed**

| #1 ((((((((((polyps[MeSH Terms]) OR colorectal polyps) OR colorectal polyp) OR colorectal polyposis) OR polyp) OR adenomatous polyposis) OR hyperplastic polyps) OR intestinal polyps) OR colonic polyps) OR polyposis coli) OR polyposis **<54,043 records>**  #2 (((((((Methylenetetrahydrofolate Reductase (NADPH2)[MeSH Terms]) OR 5,10-Methylenetetrahydrofolate reductase) OR Methylenetetrahydrofolate reductase) OR MTHFR) OR Methylenetetrahydrofolate Reductase (NADPH)) OR Methylene-THF Reductase (NADPH)) OR 5,10-Methylenetetrahydrofolate Reductase (NADPH)) OR Tetrahydrofolate Reductase, Methylene **<5,423 records >**  #3 #1 and #2  ((((((((((((polyps[MeSH Terms]) OR colorectal polyps) OR colorectal polyp) OR colorectal polyposis) OR polyp) OR adenomatous polyposis) OR hyperplastic polyps) OR intestinal polyps) OR colonic polyps) OR polyposis coli) OR polyposis)) AND ((((((((Methylenetetrahydrofolate Reductase (NADPH2)[MeSH Terms]) OR 5,10-Methylenetetrahydrofolate reductase) OR Methylenetetrahydrofolate reductase) OR MTHFR) OR Methylenetetrahydrofolate Reductase (NADPH)) OR Methylene-THF Reductase (NADPH)) OR 5,10-Methylenetetrahydrofolate Reductase (NADPH)) OR Tetrahydrofolate Reductase, Methylene) **<22 records >** |
| --- |

**WOS**

| #1 TOPIC: (polyps) OR TOPIC: (colorectal polyps) OR TOPIC: (colorectal polyp) OR TOPIC: (colorectal polyposis) OR TOPIC: (polyp) OR TOPIC: (adenomatous polyposis) OR TOPIC: (hyperplastic polyps) OR TOPIC: (intestinal polyps) OR TOPIC: (colonic polyps) OR TOPIC: (polyposis coli) OR TOPIC: (polyposis) **<45,066 records >**  #2 TOPIC: (Methylenetetrahydrofolate Reductase (NADPH2)) OR TOPIC: (5,10-Methylenetetrahydrofolate reductase) OR TOPIC: (Methylenetetrahydrofolate reductase) OR TOPIC: (MTHFR) OR TOPIC: (Methylenetetrahydrofolate Reductase (NADPH)) OR TOPIC: (Methylene-THF Reductase (NADPH)) OR TOPIC: (5,10-Methylenetetrahydrofolate Reductase (NADPH)) OR TOPIC: (Tetrahydrofolate Reductase, Methylene)  **<11,022 records >**  #3 #1 AND #2 **<83 records >** |
| --- |

**EMBASE**

| #1 'polyp'/exp OR 'haemangiopericytomatous polyp' OR 'hemangiopericytomatous polyp' OR 'polyp, haemangiopericytomatous' OR 'polyp, hemangiopericytomatous' OR 'polyps' OR 'polyposis' **<76,527 records >**  #2 'methylenetetrahydrofolate reductase (nadph2)'/exp OR '5, 10 methylenetetrahydro folate reductase (nadph)' OR 'e.c. 1.1.1.171' OR 'e.c. 1.5.1.20' OR 'methylenetetrahydrofolate reductase' OR 'mthfr' **<11,195 records >**  #3 #1 AND #2 **<48 records >** |
| --- |
